# Making best use of existing evidence when planning trials

**DOI:** 10.1186/1745-6215-12-S1-A51

**Published:** 2011-12-13

**Authors:** Verena Roloff, JPT Higgins

**Affiliations:** 1 MRC Biostatistics Unit, Cambridge, UK

## Objectives

Although meta-analyses are typically viewed as retrospective activities, they are increasingly being applied prospectively to justify planned research by providing up-to-date evidence on specific research questions. Since healthcare policy makers often base decisions on systematic reviews of reliable evidence rather than on single clinical trials, trialists should consider planning additional research with regard to the meta-analytic result.

Since adding a new study to an existing meta-analysis creates a multiple testing scenario, which standard methods do not well address, nominal significance levels need to be adjusted to preserve the overall type-I error rate. Sequential approaches to meta-analysis have been proposed, although these do not lead directly to recommendations on planning new studies.

## Methods

We propose to use the framework of adaptive clinical trial design methods to plan studies for addition to a meta-analysis.

## Results

We discuss the implementation of the adaptive method proposed by Bauer and Köhne (1994) to the meta-analysis framework, and discuss the implications of heterogeneity among studies when applying them to a random-effects meta-analysis.

By additionally deriving the conditional power of a random-effects meta-analysis under different assumptions (about the number of additional studies, their information sizes and of the heterogeneity anticipated among them), we can assess the impact of new trials on the meta-analysis. For instance, if heterogeneity is anticipated, it might not be possible for a single study to reach the desirable power no matter how large it is. Simple graphs that summarize the conditional powers of possible design alternatives provide a convenient way to explore different strategies for planning research.

We illustrate this framework for designing new trials for different scenarios using data from the Cochrane Database of Systematic Reviews.

## Conclusions

We provide a framework for designing new trials with regard to the meta-analytic result rather than the study in isolation.

